# Supplementary material for: Multidimensional stationary probability distribution for interacting active particles
Source: Sci Rep. 2015 May 29;5:10742. doi: 10.1038/srep10742 (PMC4448265; doi:10.1038/srep10742)
Supplement: Supplementary Information [file srep10742-s1.pdf]

Multidimensional Stationary Probability Distribution for Interacting Active Particles, Supplemental Material

Claudio Maggi[1],[a] Umberto Marini Bettolo Marconi[2], Nicoletta Gnan[3], and Roberto Di Leonardo[1,4]
[1]*Dipartimento di Fisica, Università di Roma "Sapienza", I-00185, Roma, Italy*
[2]*Scuola di Scienze e Tecnologie, Università di Camerino,*
Via Madonna delle Carceri, 62032, Camerino, INFN Perugia, Italy
[3]*CNR-ISC, UOS Sapienza, P.le A. Moro2, I-00185, Roma, Italy and*
[4]*CNR-IPCF, UOS Roma, Dipartimento di Fisica Università Sapienza, I-00185 Roma, Italy*

I. PROOF OF THE MAIN RESULT

Let us consider the set of stochastic differential equations:

$$\dot{x}_i = -\partial_{x_i}\Phi + \eta_i \tag{1}$$

where $i = 1, .., N$, $\partial_{x_i} = \partial/\partial x_i$ and the η_i-s are a set of independent Gaussian stochastic processes with zero mean $\langle \eta_j \rangle = 0$ and exponential time-correlation:

$$\langle \eta_i(t)\eta_j(s)\rangle = \delta_{ij}\frac{D}{\tau}e^{|t-s|/\tau}$$

The Gaussian colored-noise component η_i is necessarily produced by the Ornstein-Uhlenbeck process (Doob's theorem) [15]:

$$\dot{\eta}_i = -\frac{\eta_i}{\tau} + \frac{D^{1/2}}{\tau}\Gamma_i \tag{2}$$

where the Γ_i-s are a set of uncorrelated white-noise processes:

$$\langle \Gamma_i(s)\Gamma_j(t)\rangle = 2\delta_{ij}\delta(t-s)$$

with zero average $\langle \Gamma_i \rangle = 0$. By combining Eq.s (1) and (2) we find [15, 17]:

$$\ddot{x}_i + \frac{\partial_{x_i}\Phi}{\tau} + \sum_k \left(\frac{\delta_{ik}}{\tau} + \partial_{x_i x_k}\Phi\right)\dot{x}_k = \frac{D^{1/2}}{\tau}\Gamma_i \tag{3}$$

The Unified Colored Noise Approximation (UCNA) is done by neglecting the term $\ddot{x}_i$ in Eq. (4). With this simplification we can invert Eq. (4) to obtain

$$\dot{x}_i = -\sum_k [M^{-1}]_{ik}\frac{\partial_{x_k}\Phi}{\tau} + \frac{D^{1/2}}{\tau}\sum_k [M^{-1}]_{ik}\Gamma_k \tag{4}$$

where we have defined the matrix $M_{ij} = \delta_{ij}/\tau + \partial_{x_i x_j}\Phi$ (which is symmetric by construction being $\partial_{x_i x_k}\Phi$ the Hessian matrix) and its inverse $[M^{-1}]_{ij}$.

The Fokker-Planck equation associated with the (Stratonovich) Langevin equation (4) is given by [18]:

[a]Electronic address: `claudio.maggi@roma1.infn.it`

$$\dot{\Omega} = \sum_{l,i} \partial_{x_l} \left\{ \frac{1}{\tau} [M^{-1}]_{li} \, (\partial_{x_i} \Phi) \, \Omega \right\} + \frac{D}{\tau^2} \sum_{l,i,j} \partial_{x_l} \, \left\{ [M^{-1}]_{li} \, \partial_{x_j} \left([M^{-1}]_{ij} \, \Omega \right) \right\} \tag{5}$$

where $\Omega = \Omega(x_1, ..., x_N)$ is the probability density function. The stationary solution with vanishing current is found from Eq. (5) by setting:

$$\sum_i [M^{-1}]_{li} \, (\partial_{x_i} \Phi) \Omega + \frac{D}{\tau} \sum_{i,j} [M^{-1}]_{li} \, \partial_{x_j} \left\{ [M^{-1}]_{ji} \Omega \right\} = 0 \tag{6}$$

By multiplying Eq. (6) by M_{kl} and summing over l we get:

$$-\frac{\tau}{D} (\partial_{x_k} \Phi) \Omega - \Omega \sum_j \partial_{x_j} [M^{-1}]_{jk} = \sum_j [M^{-1}]_{jk} \, \partial_{x_j} \Omega \tag{7}$$

where we have used the identity $\sum_l M_{kl} [M^{-1}]_{li} = \delta_{ki}$. We reiterate this step by multiplying by M_{nk} and summing over k both sides of Eq. (7):

$$-\Omega \frac{\tau}{D} \sum_k M_{nk} (\partial_{x_k} \Phi) + \Omega \sum_{jk} [M^{-1}]_{jk} (\partial_{x_j} [M^{-1}]_{kn}) = \partial_{x_n} \Omega \tag{8}$$

Note that the second term on the l.h.s of Eq. (8) changes sign because

$$\partial_{x_j} (\sum_k [M^{-1}]_{jk} M_{kn}) = \partial_{x_j} (\delta_{jn}) = 0$$

that gives

$$\sum_k [M^{-1}]_{jk} (\partial_{x_j} M_{kn}) = - \sum_k (\partial_{x_j} [M^{-1}]_{jk}) M_{kn}$$

The second term on l.h.s of Eq. (8) can be further transformed if we use the identity $\partial_{x_j} M_{kn} = \partial_{x_n} M_{kj}$, which stems from the fact that M_{ij} contains the 2nd-order derivatives of the potential,:

$$\sum_{jk} [M^{-1}]_{jk} (\partial_{x_j} [M^{-1}]_{kn}) = \sum_{jk} [M^{-1}]_{jk} (\partial_{x_n} [M^{-1}]_{kj}) = \frac{1}{|M|} \partial_{x_n} |M|$$

where in the last equality $|M|$ is the determinant of the matrix M and we have used the Jacobi's formula $(1/|M|) \partial_x M = \text{Tr} \left(M^{-1} \partial_x M \right)$ [19]. Returning to Eq. (8) we thus have

$$\Omega \left(-\frac{\tau}{D} \sum_k M_{nk} (\partial_{x_k} \Phi) + \frac{1}{|M|} \partial_{x_n} |M| \right) = \partial_{x_n} \Omega \tag{9}$$

which can be integrated and gives, up to a constant multiplicative (normalization) factor :

$$\Omega = \exp \left[-\frac{\Phi}{D} - \frac{\tau}{2D} \sum_i (\partial_{x_i} \Phi)^2 \right] ||\tau M|| \tag{10}$$

where $||M||$ is the absolute value of the determinant of M. This last equation which coincides with our main result.

II. HARD REPULSIVE POTENTIAL IN $1d$: THE GCN CASE

For $d = 1$ the formula (10) reduces to the known form [15]

$$\Omega(x) = \exp\left[-\frac{\Phi(x)}{D} - \frac{\tau}{2D}|\Phi'(x)|^2\right]|1 + \tau\Phi''(x)| \tag{11}$$

To derive the form taken by Eq. (11) in presence of a steep repulsive potential we consider the fact that, in presence of such a potential, the $\Omega(x)$ strongly peaks around $x = x^*$ where $|\Phi'(x^*)| \approx \sqrt{D/\tau}$. This is intuitive since, at the point x^*, the GCN root mean-squared velocity $\sqrt{\langle\eta^2\rangle} = \sqrt{D/\tau}$ is balanced by the external (deterministic) velocity component and the particle stops. We further assume that the potential is well approximated by an exponential in the vicinity of $x = x^*$

$$\Phi \approx \lambda\sqrt{\frac{D}{\tau}}\exp[(x - x^*)/\lambda] \tag{12}$$

which consistently gives $|\Phi'(x^*)| = \sqrt{D/\tau}$. If $\Omega(x)$ is peaked around x^* this must be a maximum which gives the condition [15]

$$\Phi'''(x^*) = \frac{\Phi'(x^*)\left(\tau\Phi''(x^*) + 1\right)^2}{D\tau} \tag{13}$$

If we use Eq.s (12) and (13) into Eq. (11) and we expand it to second order around x^* we get

$$\Omega(x) \approx \exp[A + B(x - x^*)^2] \tag{14}$$

where

$$A = -\frac{\lambda\sqrt{\frac{D}{\tau}}}{D} + \log\left(\frac{\tau\sqrt{\frac{D}{\tau}}}{\lambda} + 1\right) - \frac{1}{2}$$

$$B = -\frac{7\lambda^2\tau\sqrt{\frac{D}{\tau}} + 8D\lambda\tau + 2D\tau^2\sqrt{\frac{D}{\tau}} + 2\lambda^3}{2D\lambda^3\tau + 2D\lambda^2\tau^2\sqrt{\frac{D}{\tau}}}$$

whose integral is

$$\int \Omega(x)dx = \sqrt{2\pi}\exp\left[-\frac{\lambda}{\sqrt{D\tau}} - \frac{1}{2}\right]\left(\sqrt{D\tau} + \lambda\right)^{3/2}\sqrt{\frac{D\tau}{7\lambda^2\sqrt{D\tau} + 8D\lambda\tau + 2(D\tau)^{3/2} + 2\lambda^3}} \tag{15}$$

In the limit of $\lambda \to 0$ the potential (12) approaches the potential generated by a hard wall located at x^*: $\Phi(x) = \infty$ if $x > x^*$ and $\Phi(x) = 0$ if $x < x^*$. By taking this limit the integral (15) becomes (note that $\sqrt{\pi/e} \approx 1.07$):

$$\int \Omega(x)dx = \sqrt{\frac{\pi D\tau}{e}} \approx \sqrt{D\tau} \tag{16}$$

Let us now consider one single hard wall of thickness $2x^*$ centered at $x = 0$. We also consider one GCN-driven particle that is free to move around the wall in a $1d$ space with periodic boundaries located at $\pm L/2$. Using the result given above in this case Eq. (11) becomes $\Omega = k(x - x^*) + k(x + x^*) + 1$ where $k(x - x^*) = 2\sqrt{D\tau}\delta(x - x^*)$, since, in the $\lambda \to 0$ limit, $\Phi \to 0$ and $\Omega \to 1$ whenever $|x| > x^*$ and $\Omega \to 0$ if $|x| < x^*$. By integrating Ω we get the normalization constant $\int_{-L/2}^{L/2} dx\Omega(x) = 2\sqrt{D\tau} + L^*$ and the normalized probability

$$P(x) = \frac{k(x-x^*) + k(x+x^*) + 1}{L^* + 2\sqrt{D\tau}} \tag{17}$$

where $L^* = L - 2x^*$. Eq. (17) can be used to compute the quantity $\langle|\Phi'|\rangle$ if we recall that $\Phi'(\pm x^*) = \pm\sqrt{D/\tau}$ and $\Phi'(x) = 0$ at any point $x \neq \pm x^*$. By integrating Eq. (17) we get:

$$\langle|\Phi'|\rangle = \int_{-L/2}^{L/2} dx P(x)|\Phi'(x)| = \frac{2D}{L^* + 2\sqrt{D\tau}} \tag{18}$$

III. HARD REPULSIVE POTENTIAL IN $1d$: THE RNT CASE

To show the similarity between the GCN model and "run and tumble" (RnT) model in presece of a hard potential we consider one single RnT particle moving in $1d$. In the RnT dynamics the noise consists in a two-state Poissonian process happening at rate λ [15]. The RnT noise (also known as "telegraphic" noise) is exponentially correlated in time as the GNC, but bimodally distributed switching between two velocities only: $\eta = \pm v$. The speed v and the characteristic time $\tau = (2\lambda)^{-1}$ define the diffusivity $D = v^2\tau$ of the RnT particle in absence of any external potential [6, 11]. We consider the continuity equations that can be written for the RnT model [5]:

$$\dot{L}(x) = -\partial_x \{L(x)[-v - \Phi'(x)]\} - \frac{\lambda}{2}L(x) + \frac{\lambda}{2}R(x) \tag{19}$$

$$\dot{R}(x) = -\partial_x \{R(x)[v - \Phi'(x)]\} - \frac{\lambda}{2}R(x) + \frac{\lambda}{2}L(x) \tag{20}$$

where $L(x)$ and $R(x)$ is the fraction of particles with a random velocity component pointing to the left ($\eta = -v$) and to the right ($\eta = v$) respectively. Let us now consider a hard wall as above extending between $-x^*$ and x^* with periodic boundaries at $\pm L/2$. We consider a stationary solution of Eq.s (19) and (20) of the form

$$L(x) = 2A\delta(x - x^*) + B/2 \tag{21}$$

$$R(x) = 2A\delta(x + x^*) + B/2 \tag{22}$$

where the constant A represents the fraction of particles going to the left accumulating on the wall at x^* that is equal to the fraction of particles going to the right accumulating on the wall at $-x^*$. The constant B in Eq.s (21),(22) represents the fraction of particles that are not in contact with the walls independently on the sign of their velocity. We now focus only on those particle going to the left in the neighbourhood of x^* (the same conclusions are obtained if we consider those going to the right and $-x^*$). Considering the stationary case $\dot{L} = 0$ we integrate Eq. (19) it between x^* and $x^* + \epsilon$:

$$-\{L(x^*+\epsilon)[-v + \Phi'(x^*+\epsilon)] - L(x^*)[-v + \Phi'(x^*)]\} - \frac{\lambda}{2}\int_{x^*}^{x^*+\epsilon} dx L(x) + \frac{\lambda}{2}\int_{x^*}^{x^*+\epsilon} dx R(x) = 0 \tag{23}$$

If we use Eq.s (21),(22) in Eq. (23) we get

$$-A\lambda - [\Phi'(0) - \Phi'(\epsilon)]B/2 = -A\lambda + vB/2 = 0 \tag{24}$$

where we have used the fact that $\Phi'(x^*) = -v$. Eq. (24) must be supplemented with the normalization condition

$$\int_{-L/2}^{L/2} dx\,[L(x) + R(x)] = 2A + BL^* = 1 \tag{25}$$

where $P(x) = L(x) + R(x)$ is the overall probability density and $L^* = L - x^*$. Using Eq.s (24) and (25) we find $B = \lambda/(v + \lambda L^*)$ and $A = v/[2(v + \lambda L^*)]$ that when expressed as a function of τ and D becomes

$$A = \frac{\sqrt{D\tau}}{L^* + 2\sqrt{D\tau}} \tag{26}$$

$$B = \frac{1}{L^* + 2\sqrt{D\tau}} \tag{27}$$

Using Eq.s (26) and (27) we have

$$P(x) = L(x) + R(x) = \frac{k(x - x^*) + k(x + x^*) + 1}{L^* + 2\sqrt{D\tau}} \tag{28}$$

(with $k(x) = 2\sqrt{D\tau}\delta(x)$), which is identical to Eq. (17).

IV. RADIALLY SYMMETRIC HARD POTENTIAL

Let us consider the case of a spherically symmetric potential for which Eq. (10) becomes

$$\Omega(r) = \Theta \exp\left[-\frac{\Phi(r)}{D} - \frac{\tau}{2D}|\Phi'(r)|^2\right] |1 + \tau\Phi''(r)||r + \tau\Phi'(r)|^{d-1} \tag{29}$$

where Θ is the solid angle in d-dimensions. Let us consider the case $d = 2$ ($r = \sqrt{x^2 + y^2}$, $\Theta = 2\pi$) and a hard potential that is $\Phi(r) = 0$ for $r < R^*$ and $\Phi(r) = 0$ for $r > R^*$. Let us consider one GCN-driven particle that is confined by this circular infinite-well where we expect that $\Omega(r)$ peaks strongly in correspondence of $r = R^*$. We note that Eq. (29) is identical to Eq. (11) multiplied by the term $2\pi|r + \tau\Phi'(r)|$ when $d = 2$. We further assume that multiplying Eq. (11) by $2\pi|r + \tau\Phi'(r)|$ does not change too much the shape of the peak given by the terms

$$\exp\left[-\frac{\Phi}{D} - \frac{\tau}{2D}|\Phi'|^2\right] |1 + \tau\Phi''| \approx k(r - R^*) + 1$$

where $k(r) = 2\sqrt{D\tau}\delta(r)$. In this way we have that

$$\Omega(r) \approx 2\pi[k(r - R^*) + 1]|r + \tau\Phi'(r)| \tag{30}$$

where $\Phi'(R^*) = \sqrt{D/\tau}$ and $\Phi'(r) = 0$ for $r < R^*$. The integral of $\Omega(r)$ (normalization constant) is computed by noting that, for $r < R^*$, $\Omega(r) = 2\pi r$ so that $\int_{r<R^*} dr\Omega(r) = \pi {R^*}^2$, while in the neighbourhood of R^* we get $\int_{r\approx R^*} dr\Omega(r) = 2\pi\sqrt{D\tau}(R^* + \sqrt{D\tau})$. Using these results we have from Eq. (30)

$$P(r) = \frac{2\pi[k(r - R^*) + 1]|r + \tau\Phi'(r)|}{\pi {R^*}^2 + 2\pi\sqrt{D\tau}(R^* + \sqrt{D\tau})} \tag{31}$$

From Eq. (31) we can compute:

$$\langle|\Phi'|\rangle = \int_0^{R^*} drP(r)|\Phi'(r)| = \frac{2D(R^* + \sqrt{D\tau})}{{R^*}^2 + 2D\tau + 2R^*\sqrt{D\tau}} \tag{32}$$

V. COMPARISON OF THE GCN MODEL WITH THE RNT MODEL

We briefly illustrate here how the "run and tumble" (RnT) model compares with the GCN model. The numerical results of the RnT model are compared with the theoretical results obtained by the MUCNASP showing quite unexpected analogies.

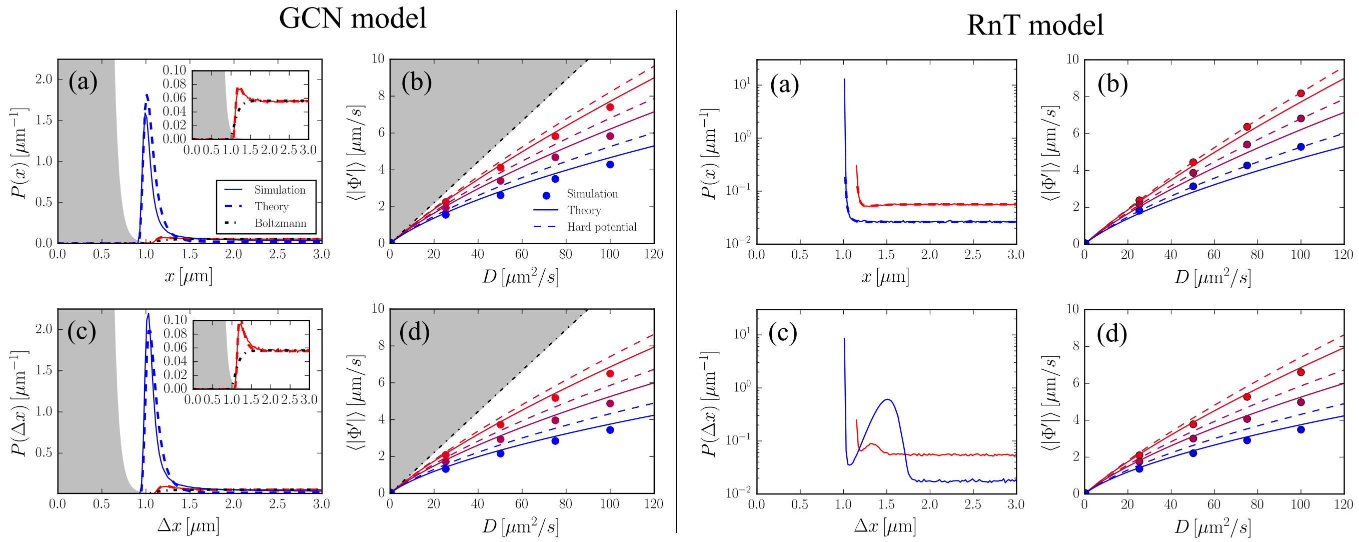

FIG. 1: **(LEFT)** GCN model **(a)** Probability density function of the position of one GCN-driven particle in presence of the external potential $\Phi = x^{-12}$ (shaded area). Full lines: simulations results, dashed lines: theory, dashed-dotted line: Boltzmann distribution. The more peaked curve corresponds to $D = 100\,\mu\text{m}^2/\text{s}$, $\tau = 1\,\text{s}$ and the less peaked one to $D = 0.4\,\mu\text{m}^2/\text{s}$, $\tau = 0.1\,\text{s}$ (zoomed in the inset) **(b)** Average value of $|\Phi'|$ as a function of D for three different values of $\tau = 0.1, 0.325, 1$ s from top to bottom respectively. Points: simulations results, full lines: theory, dashed lines: theory in the limit of an hard potential, dashed dotted line: equilibrium case with a hard potential. **(c)** Probability density function of the distance between two GCN-driven particles interacting via the potential $\Phi = \Delta x^{-12}$, same legend as Fig. (a). **(d)** Average value of $|\Phi'|$ as a function of D for three different values of $\tau = 0.1, 0.325, 1$ s from top to bottom respectively, same legend as as Fig. (b). **(RIGHT) (a)** RnT model Probability density function of the position of one RnT particle in presence of the external potential $\Phi = x^{-12}$. Full lines: simulations results, dashed lines: exact theory for the RnT (Eq. (33)). The more peaked curve corresponds to $D = 100\,\mu\text{m}^2/\text{s}$, $\tau = 1\,\text{s}$ and the less peaked one to $D = 0.4\,\mu\text{m}^2/\text{s}$, $\tau = 0.1\,\text{s}$ **(b)** Average value of $|\Phi'|$ as a function of D for three different values of $\tau = 0.1, 0.325, 1$ s from top to bottom respectively. Points: simulations results, full lines: theory (MUCNA), dashed lines: theory in the limit of an hard potential. **(c)** Probability density function of the distance between two RnT particles interacting via the potential $\Phi = \Delta x^{-12}$, same legend as Fig. (a). **(d)** Average value of $|\Phi'|$ as a function of D for three different values of $\tau = 0.1, 0.325, 1$ s from top to bottom respectively, same legend as as Fig. (b).

A. One degree of freedom

Let us start by the case in which one single particle moves in a $1d$ space of extension L (periodic boundaries are located at $\pm L/2$) in presence of the external potential $\Phi = x^{-12}$. In this case the probability distribution of the GCN driven particle peaks in correspondence of $x^* \approx 1$ as shown in Fig. 1(a)(left). This is true also for the RnT model as see Fig. 1(a)(right) where a log-scale has been used for clarity. Note that in this simple case, with one single degree of freedom, the exact $P(x)$ is know for the RnT model [6, 11, 15]:

$$P(x) = \frac{A}{D - \tau|\Phi'(x)|^2} \exp\left[\int^x dy \frac{\Phi'(y)}{D - \tau|\Phi'(y)|^2}\right] \tag{33}$$

where A is a normalization constant that absorbs the lower integration limit. Eq. (33) is plotted in Fig. 1(a)(right) as dashed line and it overlaps perfectly the numerical $P(x)$.

The average value of the absolute external velocity component $\langle|\Phi'|\rangle$ can be computed for the GCN case by using the UCNA (Eq. (11)) and is shown as full lines in Fig. 1(b)(left). However we find that this theoretical prediction for the GCN approximates well also the results of the RnT model as shown in Fig. 1(b)(right). This is somehow expected since the expression of the $P(x)$ is almost identical in the two models in the limit of a infinitely steep potential (see Eq.s (17) and (28)) represented by the dashed lines in Fig. 1(b)(left and right).

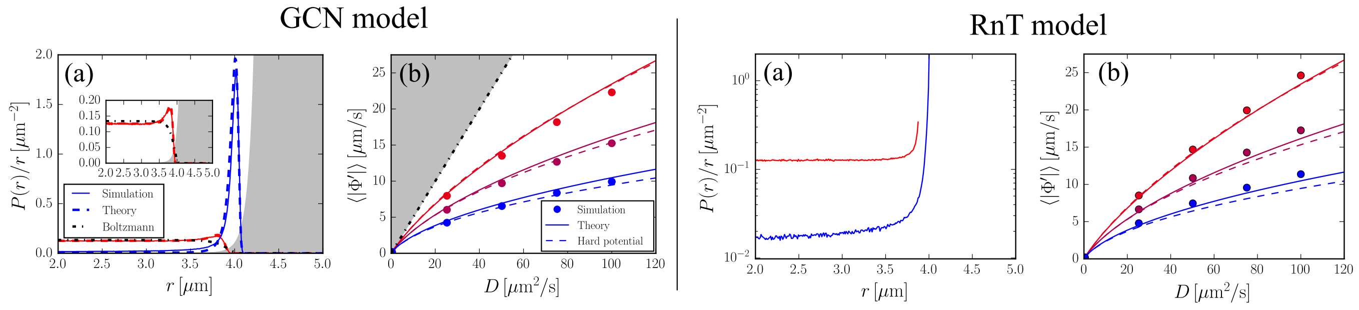

FIG. 2: **(LEFT)** GCN model **(a)** Probability density function of the radial coordinate of one GCN-driven particle in presence of the spherically symmetric external potential $\Phi = (r-R)^{-12}$ in $2d$ (shaded area). Full lines: simulations results, dashed lines: theory, dashed-dotted line: Boltzmann distribution. The more peaked curve corresponds to $D = 100\,\mu\text{m}^2/\text{s}$, $\tau = 1\,\text{s}$ and the less peaked one to $D = 0.4\,\mu\text{m}^2/\text{s}$, $\tau = 0.1\,\text{s}$ (zoomed in the inset) **(b)** Average value of $|\Phi'|$ as a function of D for three different values of $\tau = 0.1, 0.325, 1$ s from top to bottom respectively. Points: simulations results, full lines: theory, dashed lines: theory in the limit of an hard potential, dashed dotted line: equilibrium case with a hard potential. **(RIGHT)** RnT model **(a)** Probability density function of the radial coordinate of one RnT particle in presence of the spherically symmetric external potential $\Phi = (r-R)^{-12}$ in $2d$. Full lines: simulations results, dashed lines: theory. The more peaked curve corresponds to $D = 100\,\mu\text{m}^2/\text{s}$, $\tau = 1\,\text{s}$ and the less peaked one to $D = 0.4\,\mu\text{m}^2/\text{s}$, $\tau = 0.1\,\text{s}$ **(b)** Average value of $|\Phi'|$ as a function of D for three different values of $\tau = 0.1, 0.325, 1$ s from top to bottom respectively. Points: simulations results, full lines: theory (MUCNASP), dashed lines: theory in the limit of an hard potential.

B. Two particles in $1d$

We now consider two particles with coordinates x_1 and x_2 moving in $1d$ interacting via the pair potential $\Phi(\Delta x) = (x_1 - x_2)^{-12}$ with periodic boundaries at $\pm L/2$. In this case the GCN-driven particles are frequently found in contact leading to the a very peaked $P(\Delta x)$ which is well described by the MUCNASP:

$$\Omega(\Delta x) = \exp\left[-\frac{\Phi(\Delta x)}{D} - \frac{2\tau}{2D}|\Phi'(\Delta x)|^2\right]|1 + 2\tau\Phi''(\Delta x)| \tag{34}$$

as shown in Fig. 1(c)(left). Eq. (34) allows also to compute the average $\langle|\Phi'|\rangle$ that is compared with simulation results in Fig. 1(d)(left). Data compare well also with the case of a hard potential obtained by setting $\tau \to 2\tau$ in Eq. (18) (dashed lines in Fig. 1(d)(left))

Two RnT particles interacting via the same potential show a more complicated $P(\Delta x)$ that can be obtained numerically but not analytically. This is reported in Fig. 1(c)(right) where $P(\Delta x)$ show a clear secondary peak. This is qualitatively understood as follows: initially the two particle collide with opposite velocities giving rise to the peak at contact, subsequently one of the particles tumble and accelerate for a short distance (being pushed away by the potential $\Phi = \Delta x^{-12}$), when this becomes negligible the particle has moved at the distance where the 2nd peak is found: in this configuration two particles proceed together in the same direction. We note however that, despite the complicate form of the $P(\Delta x)$, the value of $\langle|\Phi'|\rangle$ obtained numerically for the RnT model is well approximated by the MUCNASP as shown in Fig. 1(d)(right). This is surprising since the $P(\Delta x)$ of the two models have a quite different form.

C. Radially symmetric potential

We consider one particle confined in a $2d$ circular potential well of the form $\Phi(r) = (r-R)^{-12}$ where $r = \sqrt{x^2+y^2}$ and $R = 5\,\mu\text{m}$. The probability density of the GCN-driven particle peaks strongly at $R^* \approx R - 1$ and this is well captured by the MUCNASP (Eq. (29)) as shown in Fig. 2(a)(left). Also the value of the radial external velocity component $\langle|\Phi'|\rangle$ obtained numerically is well described by the theory (full lines in Fig. 2(b)(left)) and it is close to the hard potential case given by Eq. (32) (dashed lines in Fig. 2(b)(left)).

Generalizing the RnT model in $2d$ we assume that the particle moves at constant speed v and the tumble randomizes completely the swimming direction (see Ref. [27]). The tumble is a Poisson process happening at rate τ^{-1} and the axial diffusion coefficient is $D = v^2\tau/2$ in absence of an external potential. The $P(r)$ of RnT particles interacting with

the potential $\Phi(r) = (r - R)^{-12}$ is shown in Fig. 2(a)(right) where we can see how the particles accumulate onto the repulsive portion of the potential. This $P(r)$ cannot be predicted theoretically and it is quite different from the $P(r)$ of the GCN-driven particles (see Fig. 2(a)(left)). From the numerical simulations we obtain the quantity $\langle|\Phi'|\rangle$ that is reported in Fig. 2(b)(right). Again, quite unexpectedly, the values of $\langle|\Phi'|\rangle$ are close to those found theoretically by the MUCNASP (full lines in Fig. 2(b)(right)) and by the same theory in the hard potential limit (dashed lines in Fig. 2(b)(right)).

[1] R. P. Feynman *Statistical Mechanics: A Set Of Lectures*, Westview Press, 2nd edition (1998)
[2] K. Huang, Statistical Mechanics (John Wiley, New York, 1987)
[3] M. E. Cates, Rep. Prog. Phys. **75** 042601 (2012)
[4] H. C. Berg, *E. coli in Motion* (Springer-Verlag, New York,2004)
[5] M. J. Schnitzer Physical Review E **48** 2553 (1993).
[6] J. Tailleur and M. E. Cates, EPL **86** 60002 (2009)
[7] J. Tailleur and M. E. Cates, Phys. Rev. Lett. **100** 218103 (2008)
[8] X. Zheng, B. ten Hagen, A. Kaiser et al. Phys. Rev. E: Stat., Nonlinear, Soft Matter Phys. **88** 032304 (2013)
[9] J. Palacci, S. Sacanna, A. P. Steinberg et al. Science **339** 936, (2013)
[10] J. R. Howse, R. A. Jones Physical review letters **99**, 048102. (2007)
[11] N. Koumakis, C. Maggi, R. Di Leonardo, Soft Matter, **10**, 5695-5701, (2014)
[12] C. Maggi, M. Paoluzzi, N. Pellicciotta, A. Lepore, L. Angelani, R. Di Leonardo, Phys. Rev. Lett., **113**, 238303, (2014)
[13] F. O'Doherty, and J. P. Gleeson. Circuits and Systems II: Express Briefs **54** : 435-439 (2007)
[14] P. Jung and P. Hänggi JOSA B **5** 979 (1988)
[15] Hänggi and P. Jung Advances in Chemical Physics **89**, 239-326 (1995)
[16] P. Jung and Hänggi Phys. Rev. A **35**, 4464 (1987)
[17] L. Cao, D. Wu and X. Luo Phys. Rev. A **47**, 57 (1993)
[18] H. Risken, The Fokker-Planck Equation: Methods of Solution and Applications (Springer, Berlin, 1984)
[19] Bellman, Richard, et al. Introduction to matrix analysis. Vol. 960. New York: McGraw-Hill, 1970.
[20] Supplemental Material
[21] L. Angelani and R. Di Leonardo Computer Physics Communications **182** 1970-1973 (2011)
[22] M. Januszewski and M. Kostur Computer Physics Communications **181** 183-188 (2010)
[23] V. A. Martinez, R. Besseling, O. A. Croze et al. Biophysical journal **103** 1637-1647 (2012)
[24] C. Maggi, A. Lepore, J. Solari, A. Rizzo and R. Di Leonardo Soft Matter **9** 10885-10890 (2013)
[25] C. Cammarota and G. Biroli. PNAS **109** 8850-8855 (2012)
S. Karmakar and G. Parisi PNAS **110** 2752-2757 (2013).
W. Kob, S. Roldán-Vargas and L. Berthier Nature Physics **8**, 164-167 (2011)
[26] L. Angelani, C. Maggi, M. L. Bernardini, A. Rizzo, R. Di Leonardo, Phys. Rev. Lett. **107** 138302 (2011)
[27] K. Martens, L. Angelani, R. Leonardo, L. Bocquet, The European Physical Journal E, **35**, 1-6, (2012)
[28] I. D. Vladescu E. J. Marsden, J. Schwarz-Linek et al. Phys. Rev. Lett. **113** 268101 (2014)
